# On some surface structures of potential taxonomic importance in families of the suborders Polydesmidea and Dalodesmidea (Polydesmida, Diplopoda)

**DOI:** 10.3897/zookeys.156.2134

**Published:** 2011-12-20

**Authors:** Nesrine Akkari, Henrik Enghoff

**Affiliations:** 1 Natural History Museum of Denmark (Zoological Museum), University of Copenhagen, Universitetsparken 15, DK-2100 København Ø – Denmark

**Keywords:** surfacemicrosculpture, prozonite, limbus, cuticular micro-scutes, ozopore, metazonital outgrowth, taxonomy

## Abstract

Surface structures have rarely been the subject of a comprehensive study in Polydesmida despite their tremendous variety within this order. A number of these peripheral structures are here studied in most families of the suborders Polydesmidea and Dalodesmidea (sensu Hoffman 1980), using scanning electron microscopy. An illustrated description of the surface sculpture of the prozonite, the limbus and the intercalary cuticular micro-scutes on the metazonite is given for the first time for the studied families, together with an account of some other poorly known surface structures. Taken together, these characters allow us to recognize two main groupings of families. The families Ammodesmidae, Cryptodesmidae, Cyrtodesmidae, Haplodesmidae, Oniscodesmidae and Pyrgodesmidae have knobs on the posterior part of the prozonites, a toothed to lobed limbus, and no micro-scutes on the metazonites, wheras the families Fuhrmannodesmidae, Polydesmidae, Dalodesmidae, Macrosternodesmidae, Nearctodesmidae, Opisotretidae and Trichopolydesmidae have no knobs on the posterior part of the prozonites, a spiky or reduced limbus, and intercalary micro-scutes on the metazonites. The results are complemented with literature records and compared with current taxonomic and phylogenetic interpretations of the group.

## Introduction

Whereas the gonopods have hitherto been acknowledged to be the most reliable source of characters for millipede identification, the details of external morphology have in most cases remained under-prospected. This is also true for the order Polydesmida although it is by far the most diverse millipede order in terms of non-gonopodal morphology. The taxonomy of several polydesmidan families, notably Fuhrmannodesmidae and Pyrgodesmidae, is in a deplorable state, and new taxonomic characters are badly needed. Rowe and Sierwald (2006) drew attention to the fact that the external morphology has been studied in only a few cases in millipedes, and gave an overview of the major works which dealt with this topic.

Scanning electron microscopy has, in many contemporary works, significantly helped to illustrate fine surface structures in millipedes. Mesibov (2009a) studied and described the several shapes of spiracles in species of the families Paradoxosomatidae and Dalodesmidae, demonstrated the great variation in the shape, location and density of the sphaerotrichomes on male legs in Dalodesmidae and noted the presence of different patterns in the integument sculpture of the metatergal tuberculation in two genera of the same family (see also Mesibov 2008). Moreover, the different arrangements and structures of the spinnerets in 16 families of Polydesmida were studied by Shear (2008), who delineated a notable variation of these structures within the studied taxa (see also Mesibov 2009a). The structure of the spinnerets was furthermore studied by Mesibov (2008, 2009a) who suggested a possible synapomorphy for some of the 10 studied dalodesmid genera even if recognizing that the use of spinneret structure in taxonomy of Polydesmida requires more sampling (see Mesibov 2009a).

The limbus, or posterior margin of the metazonites, was investigated by Schmidt (1962) who systematically described and compared the variation of shapes within numerous families, using only light microscopy.

Some polydesmidans of the families Cryptodesmidae, Haplodesmidae, and Pyrgodesmidae are earth-incrusted, i.e., adult specimens bear a coat of dirt. Shear (1973, 1977) described and illustrated special “boxes” and “branched tree-like setae” (Shear 1977) which supposedly keep the dirt on the cuticle.

During the study of a new pyrgodesmid species from Tunisia using scanning electron microscopy (Akkari and Enghoff 2011), we found a number of cuticular structures which have not hitherto been described. In order to assess their significance, we made a comprehensive survey of 22 species belonging to all the families of the suborders Polydesmidea and Dalodesmidea (see the list below and Table 1) except for the Dorsoporidae (Polydesmidea) and Vaalogonopodidae (Dalodesmidea) of which material was inaccessible for study.

**Table 1.** The studied species and the states of the three main characters examined.

	**Species**	**Knobs on the posterior surface of the prozonites**	**Limbus**	**Intercalary micro-scutes <br/> on metazonites**
**Suborder Polydesmidea**				
Ammodesmidae	*Elassystremma* sp.	+	lobes and spikes	‒
Cryptodesmidae	*Aporodesmus* sp.	‒	jagged lobes and spikes	‒
	*Elythesmus enghoffi*	+	jagged lobes and spikes	_
Cyrtodesmidae	cyrtodesmid gen. sp.	+	lobes and spikes	‒
Fuhrmannodesmidae	*Fuhrmannodesmus lividus*	–	reduced	+
	fuhrmannodesmid sp.	‒	reduced	+
	*Gyrophallus* sp.	‒	reduced	+
Haplodesmidae	*Prosopodesmus jacobsoni*	+	lobes	‒
Macrosternodesmidae	*Ophiodesmus albonanus*	‒	reduced	+
Nearctodesmidae	nearctodesmid sp.	‒	reduced	+
Oniscodesmidae	*Amphitomeus attemsi*	+	lobes and spikes	‒
Opisotretidae	*Corypholophus* sp.	‒	reduced	?+
	*Solaenaulus butteli*	–	reduced	+
Polydesmidae	*Brachydesmus superus*	‒	ramified spikes	+
	*Propolydesmus laevidentatus*	–	ramified spikes	+
Pyrgodesmidae	*Cryptocorypha ornata*	+	lobes	‒
	*Cynedesmus* sp.	+	lobes	–
	*Rharodesmus tabarkensis*	+	lobes	–
	*Tonodesmus* sp.	+	lobes	–
Trichopolydesmidae	*Napocodesmus endogeus*	‒	reduced	+
	trichopolydesmid sp.	–	reduced	+
**Suborder Dalodesmidea**				
Dalodesmidae	*Icosidesmus* sp.	‒	ramified spikes	+

Our study was mainly focused on three sets of characters: a) surface sculpture of the prozonite (anterior, cylindrical part of body ring); b) the limbus (posterior margin of body rings); c) intercalary micro-scutes on the surface of the metazonite. A few additional structures such as the cuticular outgrowths in earth-incrusted species and the ozopores are briefly presented below and compared. Moreover, recent literature accounts of 29 species from relevant families (see Table 2), including SEM illustrations or descriptions were checked and compared with our results.

**Table 2.** Literature records

	**Species**	**Reference**
**Suborder Polydesmidea**		
Ammodesmidae	*Elassystremma laeve* Vandespeigel and Golovatch, 2003. *Elassystremma prolaeve* VandenSpeigel and Golovatch, 2003	VandenSpeigel and Golovatch 2003
Cryptodesmidae	*Aporodesmus gabonicus* (Lucas, 1858)	Schmidt 1962
	*Tarmadesmus azucarensis* Kraus, 1959	
Fuhrmannodesmidae	*Fuhrmannodesmus carli* Kraus, 1955	Schmidt 1962
	*Salvadoria sagittalis* Kraus, 1954	
	*Cutervodesmus similis* Kraus, 1959	
Haplodesmidae	*Agathodesmus steeli* Silvestri, 1910	Mesibov 2009b
	*Cylindrodesmus hirsutus* Pocock, 1889	Golovatch et al. 2001
	*Eutrichodesmus armatocaudatus* Golovatch et al., 2009	Golovatch et al. 2009a
	*Eutrichodesmus basalis* Golovatch et al., 2009	
	*Eutrichodesmus communicans* Golovatch et al., 2009	
	*Eutrichodesmus inciues* Golovatch et al., 2009	
	*Eutrichodesmus similis* Golovatch et al., 2009	
Oniscodesmidae	*Oncodesmoides rectus* Kraus, 1954	Schmidt 1962
Opisotretidae	*Opisotretus kraepelini* (Attems, 1907)	Schmidt 1962
Polydesmidae	*Polydesmus complanatus* (Linneaus, 1871)	Schmidt 1962
Pyrgodesmidae	*Lobiferodesmus vanuatu* Golovatch, et al., 2008	Golovatch et al. 2008
	*Poratia digitata* (Porat, 1889)	Adis et al. 2000
	*Muyudesmus obliteratus* Kraus, 1960	
	*Cryptocorypha hoffmani* Golovatch et al. 2011	Golovatch et al. 2011
	*Myrmecodesmus hastatus* (Schubart, 1945)	Bergholz et al. 2004
	*Monachodesmus albus* Kraus, 1958	Schmidt 1962
**Suborder Dalodesmidea**		
Dalodesmidae	*Ginglymodesmus tasmanianus* Mesibov 2005	Mesibov 2009c
Not assigned to any family	*Asphalidesmus bellendenkerensis* Mesibov, 2011	Mesibov 2011
	*Asphalidesmus golovatchi* Mesibov, 2009	
	*Noteremus infimus* Mesibov, 2009	Mesibov 2009c
	*Noteremus summus* Mesibov, 2009	
	*Procophorella innupta* Mesibov, 2003	Mesibov 2003

## Materials and methods

The studied material is preserved in 70 % ethanol and deposited in the Natural History Museum of Denmark (Zoological Museum, University of Copenhagen, ZMUC). Earth-incrusted specimens were cleaned for scanning electron microscopy by soaking in a solution of commercial detergent (©Biotex) and/or by ultrasound then air dried. SEM pictures were made with a JEOL JSM-6335F scanning electron microscope, then processed and assembled with Adobe Photoshop CS5 software.

When not otherwise indicated, we have followed the classification proposed by Hoffman (1980) as updated by Shelley (2003).

### Studied specimens

**Suborder Polydesmidea Pocock, 1887**

Ammodesmidae Cook, 1896

*Elassystremma* sp., Tanzania, Unzungwa Mts, Iringa Region, Uzungwa Scarp Forest Res., above Chita village, 1600–1650m, 8–13.ix.1984, pitfall Traps in Montane Rain Forest, N. Scharff leg. (ZMUC 00020487).

Cryptodesmidae Karsch, 1879

*Aporodesmus* sp., female, Cameroun, Northwest Province, Menchum Div. Near L. Oku forest, in litter, ca. 2150m, N6°12', E10°27', 7–13.ii.1992, C. Griswold, S. Larcher, N. Scharff and C. Wanzie leg. (ZMUC 00020478).

*Elythesmus enghoffi* Hoffman, 1978, female, Tanzania, W. Usambara Mts, Mazumbai Forest Reserve, 19–29.ix.1992, M. Andersen leg. (ZMUC 00020477).

Cyrtodesmidae Cook, 1896

Cyrtodesmid sp., female, Colombia, Páramo de Sumapaz, soil under grasses, 3600m, 5.x.1978, H. Sturm leg. (ZMUC 00020494).

Fuhrmannodesmidae Brölemann, 1916

*Fuhrmannodesmus lividus* Carl, 1914, male, Colombia, Par de Monserrate, near Bogotá, 3250m, dead leaves of *Espeletia grandifolia*, 18.iv.1969, H. Sturm (ZMUC 00020483).

fuhrmannodesmid sp. (Arndt et al. 2008), female, Spain, Canary Islands, La Palma, Pared Vieja, 21.ii.-5.iii.2002, E. Arndt leg. (ZMUC 00020492).

*Gyrophallus* sp., female, Colombia, 1991, H. Sturm leg. (ZMUC 00020484).

Haplodesmidae Cook, 1895

*Prosopodesmus jacobsoni* Silvestri, 1910, female, Fiji Isl, Viti Levu Suva, in garden, 2–3.ix.1995, A van Hart leg. (ZMUC 00020476).

Macrosternodesmidae Brölemann, 1916

*Ophiodesmus albonanus* (Latzel, 1895), male, Denmark, NE Zaland, Copenhagen, Utterslev Mose, 22.iv.1973, H. Enghoff leg. (ZMUC 00020491).

Nearctodesmidae Chamberlin and Hoffman, 1950

nearctodesmid sp., male, Calif. Humboldt Co. Jolly Giant Canyon, 300–650m, Arcata Comm. For 13.i.1979, A.K. Johnson, R. M. Shelley leg. (ZMUC 00020482).

Oniscodesmidae de Saussure, 1860

*Amphitomeus attemsi* (Schubart, 1934), female, Dania: Nez UB47, Copenhagen, Botanical garden, væksthus, 16.iv.1986, H. Enghoff and Z. Korsòs leg. (ZMUC 0002046).

Opisotretidae Hoffman, 1980

*Corypholophus* sp., female, Thailand, Chieng Mai Province, Doi Inthanon N. P., Mae Ya, 6–700m (ZMUC 00020479).

*Solaenaulus butteli* (Carl, 1922), male, Fiji Isl. Viti Levu Suva, in garden, 2–3.ix.1995, A van Harten leg. (ZMUC 00020480).

Polydesmidae Leach, 1815

*Brachydesmus superus* (Latzel, 1884), female, Tunisia, NW, Jendouba Governorate, 9km of Hammam Bourguiba (West of Aïn Draham), N36°48.046, E08°39.544, 379m, Pine Forest, close to river, under stones, logs and leaf litter, 22.iii.2008, P. Stoev and N. Akkari leg. (ZMUC 00020496).

*Propolydesmus laevidentatus* (Loksa, 1967), male, Madeira, Faja da Nogueria, N. Side, ca. 800m. a.s.l. *Laurisilva* with *Ocoteas*, 20.xi.1980, H. Enghoff and O. Martin leg. (ZMUC 00020475).

Pyrgodesmidae Cook, 1895

*Cryptocorypha ornata* (Attems, 1938), unsexed fragment, Hawaii Isl., Kauai, in a grotto, moss and *Adiantum*, 20.x.1962, M. Hammer leg. (ZMUC 00020493)

*Cynedesmus* sp., female, La Gomera, Valle Gran Rey, litter, in banana plantation, 10m, 2.xii.1987, A. Fjellberg leg. (ZMUC 00020485).

*Rharodesmus tabarkensis* Akkari and Enghoff, 2011, male, Tunisia, NW, Jendouba Governorate, Tabarka, N36°58'10.5", E8°45'35.6", alt. < 40m, coastal slope below the Genoese fort, under stones, 9.iii.2009, N. Akkari and H. Enghoff leg. (ZMUC 00020532).

*Tonodesmus* sp., male, Spain, Almeria Sima terminal, T. M. Eidijo, 28.v.2000, M. Piquer and J.G. Pedro leg. (ZMUC-00020495).

Trichopolydesmidae Verhoeff, 1910

*Napocodesmus endogeus* Ceuca, 1974, female, Moldavian SSR, Tiraspol, deep in orchard soil, 1985, S. Golovatch leg. (ZMUC 00020481).

trichopolydesmid sp. Slovakia, Slovak Karst, Ardouská Cave A-04-47, 5.x.2004, A. Mock leg. (ZMUC 00020490).

**Suborder Dalodesmidea Hoffman, 1980**

Dalodesmidae Cook, 1896

*Icosidesmus* sp., male, New Zealand, South Isl, Christchurch Banks Peninsula, Hinewai Reserve, Big Kanuka Trail, 3 iii.2010, S43°48'38.0", E173°01'15.6", 508m, sifted leaf litter and mosses, N. Scharff and G. Hormiga leg. (ZMUC 00020488).

## Results

### Fine sculpture of the prozonite

Figs 1–9

The prozonite of the studied species is divided into two main parts separated by a transverse ridge. While the anterior part is rather uniform, showing a scaly aspect, the posterior surface displays varied patterns within the studied families.

**Figures 1–6.**
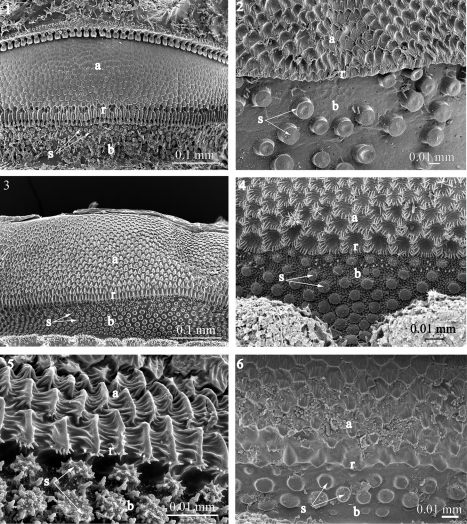
Fine sculpture of the prozonite in the families Ammodesmidae, Cryptodesmidae, Cyrtodesmidae, Haplodesmidae, Oniscodesmidae and Pyrgodesmidae **1** *Elassystremma* sp. **2** *Elythesmus enghoffi*, **3** cyrtodesmid sp. **4** *Prosopodesmus jacobsoni* **5** *Amphitomeus attemsi* **6** *Rharodesmus tabarkensis*. Abbreviations: **a** anterior part of the prozonite, **b** posterior part of the prozonite, **r** ridge, **s** spherical knobs.

**Figures 7–9.**
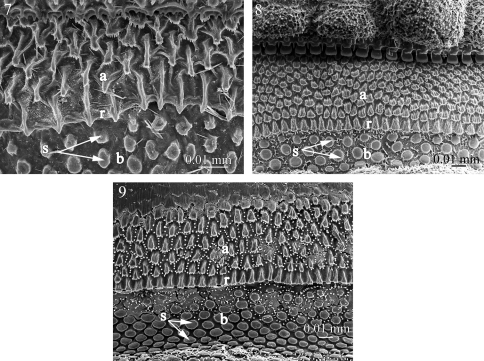
Fine sculpture of the prozonite in pyrgodesmid species. **7** *Tonodesmus* sp. **8** *Cynedesmus* sp. **9** *Cryptocorypha ornata*. Abbreviations: **a** anterior part of the prozonite, **b** posterior part of the prozonite, **r** ridge, **s** spherical knobs.

In the examined species of Ammodesmidae, Cryptodesmidae, Cyrtodesmidae, Haplodesmidae, Oniscodesmidae and Pyrgodesmidae the anterior part of the prozonite (a) displays a covering of small scaly units, juxtaposed in series of irregular transverse rows, becoming elongated in the posteriormost row to form a transverse ridge (r) marking the border of this part (Figs 1–9). In front of the ridge, the general aspect is quite uniform in all the above cited families, with lozenge-shaped units. These units could sometimes be star-like, furrowed, and marginally jagged (cyrtodesmid sp.) (Fig. 3). In *Amphitomeus attemsi* (Oniscodesmidae), the units are more elongated and strongly prominent, interconnected with parallel cuticular ridges (Fig. 5). On the other hand, the microsculpture of the anterior part of the prozonite in *Prosopodesmus jacobsoni* (Haplodesmidae) takes the shape of hollow chambers separated by walls of “microvilli-like” structures (Fig. 4). The posterior surface of the prozonite (b) is, in all examined species of these six families, characterized by a regular covering of sub-spherical knobs (s) placed on a smooth to microgranulated background. The cover of knobs is regularly dense in most cases (Figs 1, 3–9)though fairly dispersed in *Elythesmus enghoffi* (Cryptodesmidae) (Fig. 2). These knobs are generally uniformly smooth (Figs 1–3, 6–9) but sometimes exhibit special configurations: lobed in *Prosopodesmus jacobsoni* and spiky in *Amphitomeus attemsi* (Figs 4, 5).

**Figures 10–13.**
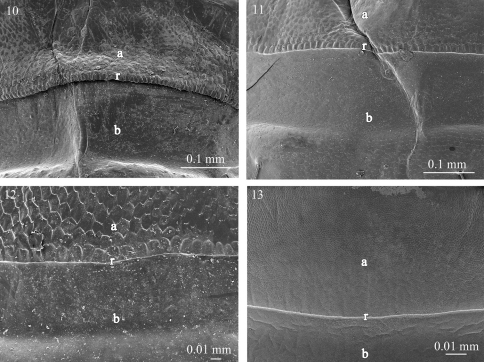
Fine sculpture of the prozonite in **10** *Fuhrmannodesmus lividus* (Fuhrmannodesmidae) **11** *Gyrophallus* sp. (Fuhrmannodesmidae) **12** *Propolydesmus laevidentatus* (Polydesmidae) **13** *Icosidesmus* sp. (Dalodesmidae). Abbreviations: **a** anterior part of the prozonite, **b** posterior part of the prozonite, **r** ridge.

**Figures 14–17.**
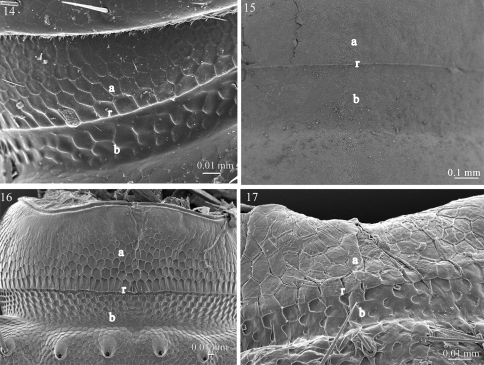
Fine sculpture of the prozonite in **14** *Ophiodesmus albonanus* (Macrosternodesmidae) **15** nearctodesmid sp. (Nearctodesmidae) **16** *Solaenaulus butteli* (Opisotretidae) **17** *Napocodesmus endogeus* (Trichopolydesmidae). Abbreviations: **a** anterior part of the prozonite, **b** posterior part of the prozonite, **r** ridge.

The examined species of the families Fuhrmannodesmidae, Polydesmidae, Dalodesmidae, Macrosternodesmidae, Nearctodesmidae, Opisotretidae and Trichopolydesmidae show an anterior surface of the prozonite with polygonal units serrated marginally. However, these units are much less conspicuous and prominent than in the species of the first set of families (Figs 10–17). The posterior border of the anterior part of the prozonite is similarly marked by a transverse ridge but its units are nevertheless only slightly modified. The posterior surface of the prozonite remarkably differs by the complete absence of the sub-spherical knobs described above; instead the surface is smooth to scaly (Figs 10–17).

### The limbus

Figs 18–34

**Figures 18–22.**
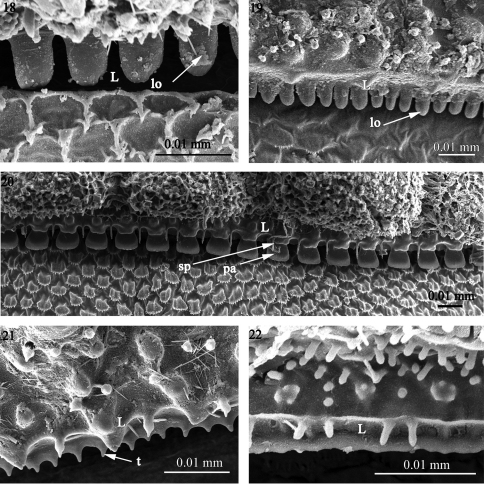
The structure of the limbus in **18** *Prosopodesmus jacobsoni* (Haplodesmidae) **19** *Rharodesmus tabarkensis* (Pyrgodesmidae) **20** *Cynedesmus* sp. (Pyrgodesmidae) **21** *Tonodesmus* sp. (Pyrgodesmidae) **22** *Cryptocorypha ornata* (Pyrgodesmidae). Abbreviations: **L** limbus, **lo** lobe, **pa** palette-like lobe, **sp** spike, **t** tooth-like lobe.

**Figures 23–26.**
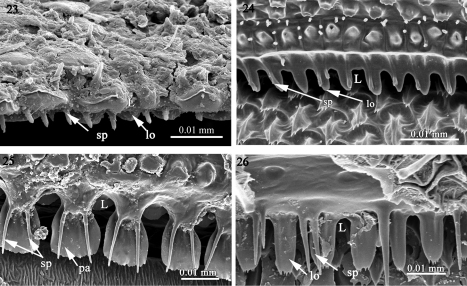
The structure of the limbus in **23** *Amphitomeus attemsi* (Oniscodesmidae) **24** cyrtodesmid sp. (Cyrtodesmidae) **25** *Elassystremma* sp. (Ammodesmidae) **26** *Elythesmus enghoffi* (Cryptodesmidae). Abbreviations: **L** limbus, **lo** lobe, **sp** spike, **pa**: palette-like lobe.

**Figures 27–30.**
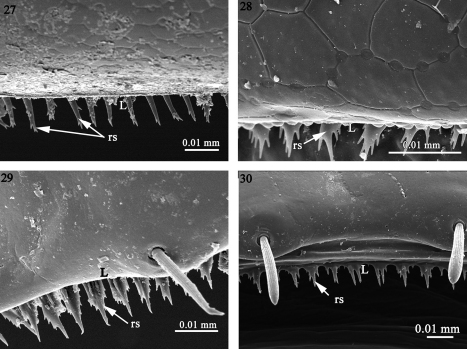
The structure of the limbus in **27** *Icosidesmus* sp. (Dalodesmidae), **28** *Ophiodesmus albonanus* (Macrosternodesmidae) **29** *Propolydesmus laevidentatus* (Polydesmidae) **30** *Brachydesmus superus* (Polydesmidae). Abbreviations: **L** limbus, **rs** ramified spike.

**Figures 31–34.**
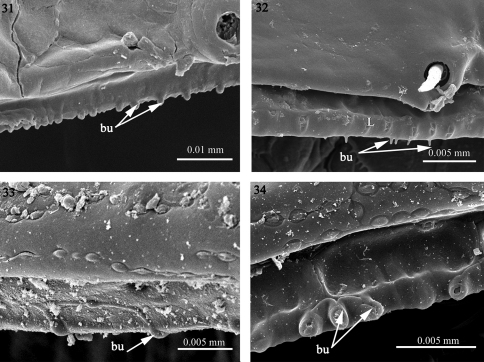
The structure of the limbus **31** *Solaenaulus butteli* (Opisotretidae) **32** *Napocodesmus endogeus* (Trichopolydesmidae), **33** *Fuhrmannodesmus lividus* (Fuhrmannodesmidae) **34** fuhrmannodesmid sp. (Fuhrmannodesmidae). Abbreviations: **bu** bulges, **L** limbus.

The limbus (L) displays three major patterns of shapes in the studied families:

1) A regular set of rounded lobes (lo) placed in one row as in *Prosopodesmus jacobsoni*(Haplodesmidae), *Rharodesmus tabarkensis*(Pyrgodesmidae) (Figs 18, 19) or two superposed rows of 'palette-shaped' lobes (pa) separated by spikes (sp) as in *Cynesdesmus* sp. (Pyrgodesmidae) (Fig. 20) although in some species of the latter family (e.g. *Tonodesmus* sp. and *Cryptocorypha ornata*) the lobes are more tooth-like (t) (Figs 21, 22). The lobes are also surmounted by fine spikes as in *Amphitomeus attemsi* (Oniscodesmidae), cyrtodesmid sp. (Figs 23, 24), *Elassystremma* sp. (Ammodesmidae) in which the lobes are moreover stocky or ‘palette-like’(pa) and serrated (Fig. 25) and in *Elythesmus enghoffi*(Cryptodesmidae) where the spikes are more elongate and inserted between the jagged lobes (Fig. 26).

2) A series of ramified spikes (rs) in *Icosidesmus* sp. (Dalodesmidae) (Fig. 27) or “icicles” in *Ophiodesmus albonanus* (Macrosternodesmidae), *Propolydesmus laevidentatus* and *Brachydesmus superus* (Polydesmidae) (Figs 28–30).

3) Reduced. In *Solaenaulus butteli* (Opisotretidae), *Napocodesmus endogeus* (Trichopolydesmidae) and *Fuhrmannodesmus lividus* (Fuhrmannodesmidae) the limbus is hardly developed, taking the shape of a regular edge bearing a few scattered bulges (bu) which could be isolated or grouped, e.g. sets of three bulges in furhmannodesmid sp. (Figs 31–34).

### A peculiar structure of the metazonites: intercalary cuticular micro-scutes

Figs 35–41

**Figures 35–41.**
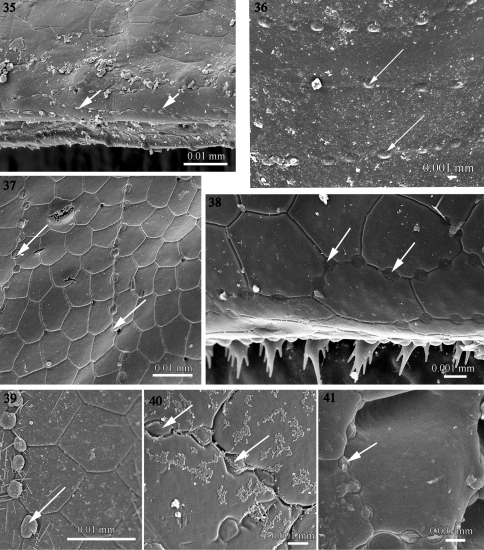
Intercalary micro-scutes on the metazonitesof **35** *Fuhrmannodesmus lividus* (Fuhrmannodesmidae) **36** *Propolydesmus laevidentatus* (Polydesmidae) **37** *Icosidesmus* sp. (Dalodesmidae) **38** *Ophiodesmus albonanus* (Macrosternodesmidae) **39** nearctodesmid sp. (Nearctodesmidae) **40** *Solaenaulus butteli* (Opisotretidae) **41** *Napocodesmus endogeus* (Trichopolydesmidae). Arrows point to the micro-scutes.

The studied species of the families Fuhrmannodesmidae, Polydesmidae, Dalodesmidae, Macrosternodesmidae, Nearctodesmidae, Opisotretidae and Trichopolydesmidae (cf. Table 1) present a peculiar structure on the metazonital surface: between the normal polygonal cuticular scutes which cover the metazonital surface there are rows of small ovoid “intercalary scutes”. The placement of these structures is unlikely to be accidental or indicating any kind of bacterial infection as they seem well arranged in a regular pattern, appearing like spaced nodes or pearls aligned on strings crossing the surface of the metazonites (Figs 35–41). These structures have never been documented. However, they are visible on an illustration in Mesibov [2003, fig. 3, (right)], for *Procophorella innupta* Mesibov, 2003 (Dalodesmidea).

### Some other poorly known surface structures

Figs 42–49

**Figures 42–43.**
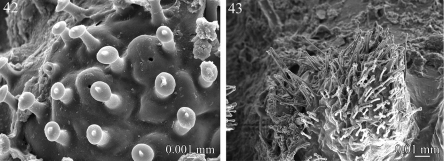
Cuticular outgrowths **42** *Rharodesmus tabarkensis* (Pyrgodesmidae) **43** *Elassystremma* sp. (Ammodesmidae).

**Figures 44–49.**
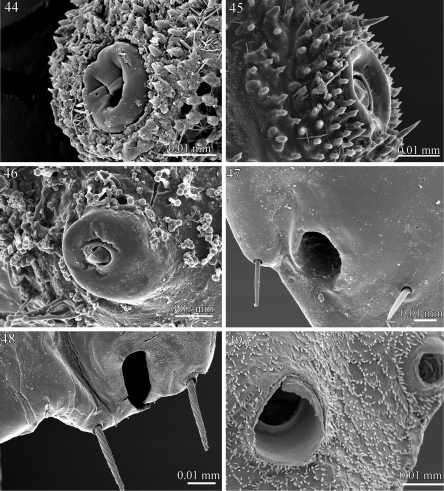
Ozopores **44** *Rharodesmus tabarkensis* (Pyrgodesmidae) **45** *Tonodesmus* sp. (Pyrgodesmidae) **46** *Elassystremma* sp. (Ammodesmidae) **47** *Propolydesmus laevidentatus* (Polydesmidae) **48** *Gyrophallus* sp. (Fuhrmannodesmidae) **49** *Corypholophus* sp. (Opisotretidae).

The surface structure of most earth-incrusted species is characterized by the presence of papilla-like cuticular outgrowths which are particularly abundant in Ammodesmidae, Cyrtodesmidae and Pyrgodesmidae. These papillae are boletiform (mushroom-shaped) and are presumed to keep the cuticular secretions and adhering soil particles in place. Displaying variation in length, shape of the apex, and density on the surface, the papillae are short, with a rounded apex in *Rharodesmus tabarkensis* (Fig. 42) and *Cynedesmus* sp., elongate and slender in *Elassystremma* sp. (Fig. 43) and in cyrtodesmid sp. The same structures have been mentioned in previous works, generally quoted as “papillis” (Silvestri 1925, 1947), “Papillen” (Attems 1940), or “microvilli” in most of Golovatch’s works ‒ illustrations can be found in Golovatch et al. (2009a, figs 24A, B, E, F). Shear (1977) was the first to provide SEM illustrations of such structures and to comment on their possible function.

The ozopores (defense gland openings) display a notable variation within the examined families. In *Prosopodesmus jacobsoni* (Haplodesmidae), *Tonodesmus* sp., *Rharodesmus tabarkensis*, *Cynedesmus* sp. (Pyrgodesmidae) and *Elassystremma* sp. (Ammodesmidae), they open on small rounded discs, situated on the surface of the paratergites or on porosteles. The discs are of variable thickness and diameter, bear an apparent internal closing mechanism and are externally bordered with several whorls of papillae (Figs 44–46). On the other hand, the ozopores appear as simple sub-circular openings on the surface of the paratergites in *Icosidesmus* sp. (Dalodesmidae), *Propolydesmus laevidentatus* (Polydesmidae), *Fuhrmannodesmus lividus* and *Gyrophallus* sp. (Fuhrmannodesmidae), and *Corypholophus* sp. (Opisotretidae) (Figs 47–49).

## Discussion

Polydesmida is the most speciose millipede order, and despite the fact that it has remained quite stable in terms of number of families (Shelley 2003), its taxonomy is far from being satisfactory. In his attempt to classify the suborders Polydesmidea and Dalodesmidea, Hoffman (1980: 146) expressed his dissatisfaction and pessimism: “The groupings set forth in the following pages are to a large extent exercises in futility, but may have some reference value in a bibliographic sense”. Nevertheless, Hoffman’s (1980) main classification scheme still stands and has been adopted by most subsequent authors. Simonsen (1990), studying the phylogeny of Polydesmida, made a number of changes and synonymies. Notably, Simonsen (1990) placed Dalodesmidae + Vaalogonpodidae as sister-group of Polydesmidae, i.e., the suborder Dalodesmidea nested within the suborder Polydesmidea. However, most of Simonsen’s conclusions were soon after criticized by several authors because they were based on insufficient data and bold assumptions (e.g. Golovatch 1996, Shelley 2003).

Taken together, the fine sculpture of the prozonite, the structure of the limbus and the presence/absence of metazonital micro-scutes indicate two main groupings of families within the suborders Polydesmidea and Dalodesmidea. The first group (A) comprises the families Ammodesmidae, Cryptodesmidae, Cyrtodesmidae, Haplodesmidae, Oniscodesmidae and Pyrgodesmidae. These six families have a cover of sub-spherical knobs on the posterior surface of the prozonites (Figs 1–9) and a toothed limbus constituted of a series of lobes which may or not be surmounted by spikes (Figs 18–26).

The second group (B) encompasses the families Fuhrmannodesmidae, Polydesmidae, Dalodesmidae Macrosternodesmidae, Nearctodesmidae, Opisotretidae and Trichopolydesmidae, and is characterized by 1) an absence of knobs on the posterior surface of the prozonite which is smooth to scaly (Figs 10–17), 2) an absence of lobes on the limbus which has instead a jagged margin or spikes with varied complexity (Figs 27–34), and 3) intercalary micro-scutes on the surface of the metazonites (Figs 35–41), absent in the first set of families.

An assessment of several recent species descriptions and SEM illustrations shows in most cases similar structural arrangements of the prozonites. The presence of sub-spherical knobs on the posterior surface of the prozonite has been verified in a few additional genera and species of Pyrgodesmidae, such as *Lobiferodesmus vanuatu* (Golovatch et al. 2008, fig. 4C), *Poratia digitata* (Adis et al. 2000, figs 25, 26, 27; Golovatch and Sierwald 2000, figs 1, 6), *Poratia* (*= Muyudesmus*) *obliterata* (Adis et al. 2000, fig. 29) and *Cryptocorypha hoffmani* (Golovatch et al. 2011, fig. 38). Moreover, comparable structures are seen in the ammodesmids *Elassystremma proleave* and *Elassystremma leave* (VandenSpeigel and Golovatch 2003, figs 7, 9, 18) and for the haplodesmid *Agathodesmus steeli* (Mesibov 2009b, figs 4B, 5C, 6C). However, we noticed some differences in *Eutrichodesmus basalis* (Golovatch et al. 2009a, figs 1C, 3C), *Eutrichodesmus armatocaudatus* (Golovatch et al 2009a fig. 6F), *Eutrichodesmus communicans* (Golovatch et al. 2009a fig. 11F) and *Eutrichodesmus incisus* (Golovatch et al 2009a, fig. 22E) although low image resolution doesn’t allow us to draw any conclusion about these species, neither about those cited in Golovatch et al. (2009b).

The fine structure of the prozonite in both species of the Tasmanian dalodesmid genus *Noteremus*, *Noteremus summus* and *Noteremus infimus* (Mesibov 2009c, fig. 3) perfectly agrees with what we recorded in the dalodesmid *Icosidesmus* sp. (Fig. 13). The genus *Asphalidesmus*, in contrast, exhibits a pyrgodesmid-like pattern with a conspicuous cover of sub-spherical knobs on the posterior surface of the prozonite. In his description of *Asphalidesmus golovatchi*, Mesibov (2009c) wrote “prozonites with narrow band of longitudinal ridges just anterior to suture, elsewhere uniformly covered with very small protuberances with blunt, rounded tips directed slightly posteriorly”. Mesibov (2011) did later describe several new species of the same genus on which the posterior prozonite protuberances were visible, especially on *Asphalidesmus bellendenkerensis* Mesibov, 2011 (Mesibov 2011, fig. 4A).

Although assigned to Dalodesmidea (Mesibov 2009c), the similarity of the genus *Asphalidesmus* to the first set of families (Ammodesmidae, Cryptodesmidae, Cyrtodesmidae, Haplodesmidae, Oniscodesmidae and Pyrgodesmidae) might not be a surprise knowing that the taxonomic position of *Asphalidesmus* has been matter of controversy. In fact, the genuswas originally described in Dalodesmidae then subsequently placed in Fontariidae, Vanhoeffeniidae, and Haplodesmidae, listed in Xystodesmidae, and assigned to Cryptodesmidae and Oniscodesmidae (see Mesibov 2009c). Despite lacking sphaerotrichomes and showing a few further peripheral structures resembling more Pyrgodesmidae than Dalodesmidae and Vaalogonopodidae, *Asphalidesmus* was informally placed in the suborder Dalodesmidea (see Golovatch 2003). This was subsequently confirmed by Mesibov (2009c) although both authors agree in not assigning the genus to any family (Golovatch 2003, Mesibov 2009c).

Several literature records clearly support the patterns described above for the limbus. For example, the limbus shows a series of lobes with or without additional spikes in the pyrgodesmid species *Lobiferodesmus vanuatu* (Golovatch et al. 2008, fig. 4D) and *Myrmecodesmus hastatus* (Bergholz et al. 2004, figs 5, 6), the ammodesmids *Elassystremma prolaeve* and *Elassystremma laeve* (VandenSpeigel and Golovatch 2003, figs 7, 18), and the haplodesmids *Agathodesmus steeli* (Mesibov 2009b, fig. 6C), *Cylindrodesmus hirsutus* (Golovatch et al. 2001, fig. 11) and *Eutrichodesmus similis* (Golovatch et al. 2009a, fig. 19E). The limbus is, on the other hand, composed of a number of ramified spikes in, for example, the dalodesmid *Ginglymodesmus tasmanianus* (Mesibov 2005, fig. 4A). Schmidt (1962, figs 22–29) provided detailed descriptions and drawings of the limbus for a number of Polydesmida species *viz*. *Oncodesmoides rectus* (Oniscodesmidae), *Polydesmus complanatus* (Polydesmidae), *Opisotretus kraepelini* (Opisotretidae), *Aporodesmus gabonicus*, *Monachodesmus albus* (Pyrgodesmidae), *Tarmadesmus azucarensis* (Cryptodesmidae), *Fuhrmannodesmus carli*, *Salvadoria sagittalis*, and *Cutervodesmus similis* (Fuhrmannodesmidae). In all these species, the limbus is strikingly similar to what we observed in the studied species belonging to the same families.

**Figures 50–51.**
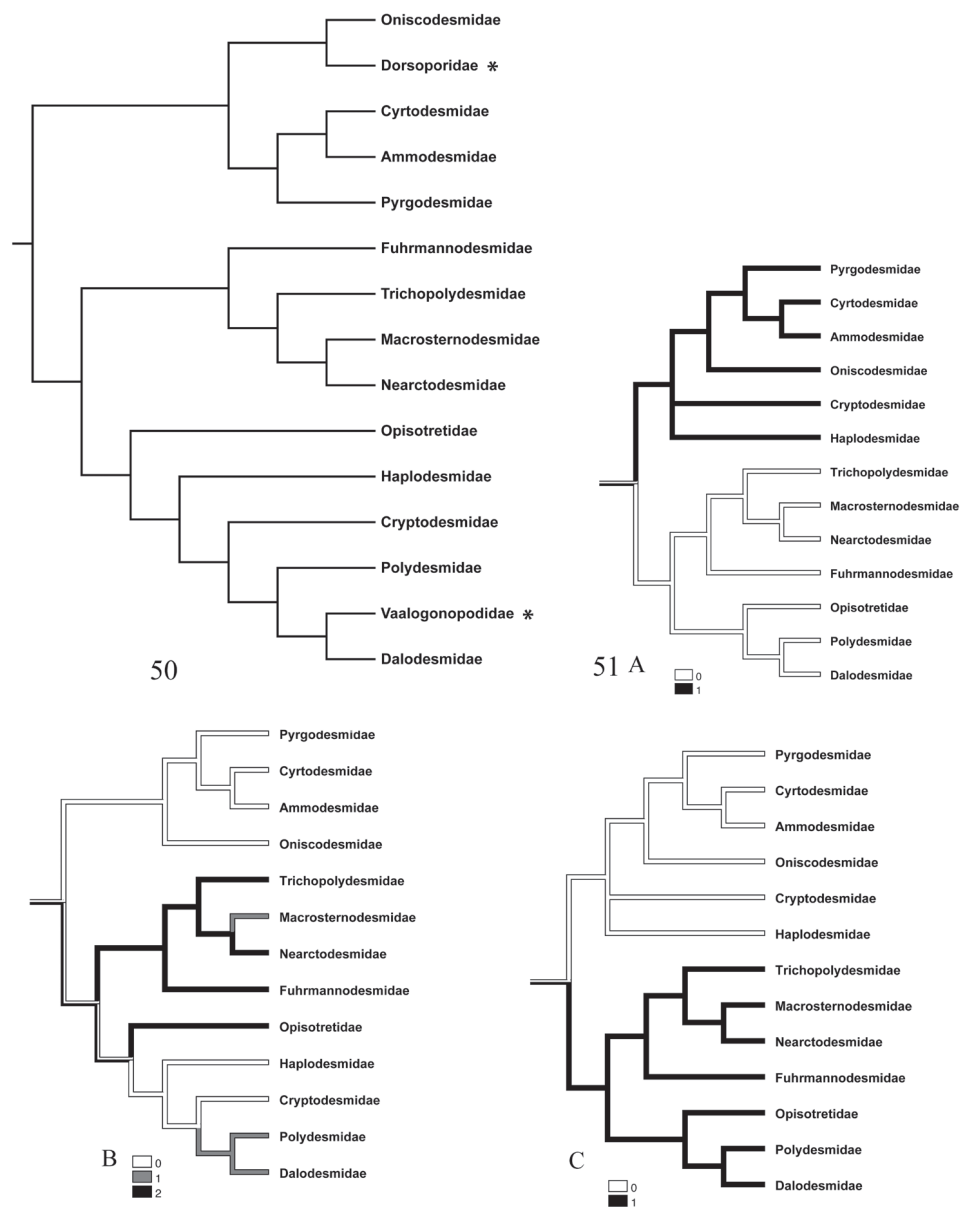
**50** Family-level cladogram of suborders Polydesmidea + Dalodesmidea according to Simonsen (1990). Haplodesmidae here corresponds to Haplodesmidae + Doratodesmidae on Simonsen’s original cladogram; families not studied here are marked with asterisks **51** Branching diagrams (not cladograms) based on Fig. 50 but modified to illustrate the distribution of the different states of the three studied characters: A. presence of knobs on the anterior part of the prozonite, B. shape of the limbus, C. presence of intercalary micro-scutes on the metazonites (see Appendix for character states).

Though we are aware that the present data alone do not warrant a strict cladistic analysis, we have compared our findings (Fig. 51) with the only existing phylogenetic work on the order Polydesmida (Simonsen 1990). The two main groups (A) and (B) mentioned above agree remarkably well with the basal dichotomy in Simonsen’ cladogram (Fig 50), except for the families Haplodesmidae and Cryptodesmidae which belong to our group (A) but which according to Simonsen are in the clade which otherwise includes our group (B). However, the general knowledge about these two families and their diversity is still incomplete, and no evidence has hitherto been provided in support of their monophyly. In his revision of the Haplodesmidae, Golovatch (2009a) recognized two major ‘grades’, the haplodesmid and doratodesmid grades, according to their somatic (non-sexual) characters and capacity for “volvation” (coiling into a sphere). In the present study, only the haplodesmid genus *Prosopodesmus* was studied. Golovatch et al. (2009a) characterised *Prosopodesmus* as a “pyrgodesmid-like haplodesmid” and considered it as basal in the Haplodesmidae (together with *Rhipidopeltis* Miyosi, 1958).

**Figures 52–53.**
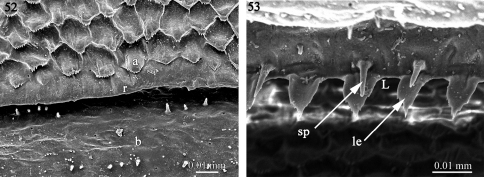
Prozonite and limbus in *Aporodesmus* sp. (Cryptodesmidae) **52** Prozonite fine sculpture **53** The limbus. Abbreviations: **a** anterior part of the prozonite, **b** posterior part of the prozonite, **L** limbus, **le** dentate leaf-shaped element of the limbus, **r** ridge, **sp** spike.

The Cryptodesmidae studied by us alos present a complication as *Elythesmus* has knobs on the posterior part of the prozonites (Fig. 2) and a lobed limbus (Fig. 26) as in families group A, whereas *Aporodesmus* has a posterior prozonite surface free of knobs (Fig. 52) and dentate leaf-shaped (le) elements and spikes (sp) on the limbus (Fig. 53).

As stated above, the second set of families (B) have no lobes on the limbus. Instead, ramified spikes and “icicles” (Figs 27–30) can be observed in the families Dalodesmidae, Macrosternodesmidae and Polydesmidae, which clearly separates them from the Fuhrmannodesmidae, Trichopolydesmidae, Nearctodesmidae and Opisotretidae in which the limbus is hardly developed and bears but few scattered bulges (Figs 31–34). However, this separation fits neither with Hoffman’s (1980) classification in which the Macrosternodesmidae, Nearctodesmidae, Trichopolydesmidae and Fuhrmannodesmidae form the superfamily Trichopolydesmoidea (see also Shelley 2003), nor with the phylogenetic analysis of Simonsen (1990) in which the Macrosternodesmidae and Nearctodesmidae appear in the same clade while the Opisotretidae, a separate clade, is grouped with a different set of families (Fig. 50). The simple limbus could well be a plesiomorphic character state expressed in the Fuhrmannodesmidae, Trichopolydesmidae, Nearctodesmidae and Opisotretidae (see also Simonsen 1990).

In the present work, we do not pretend to offer a new subordinal classification of Polydesmida, or to solve any of the taxonomic problems related to families – a colossal task which definitely will require a lot more sampling and character scoring, including the gonopod structures which haven’t been considered here. However, documenting these remarkable surface structures and trying to compare them between the different families will perhaps contribute to bringing new insights, leading towards a better understanding of polydesmidean and dalodesmidean relationships.

## Figures and Tables

**Character matrix:** Only the species studied by us have been coded.

	**Char. no.**	**1**	**2**	**3**
Suborder Polydesmidea Pocock, 1887				
Ammodesmidae Cook, 1896	*Elassystremma* sp.	1	0	0
Cryptodesmidae Karsch, 1879	*Elythesmus enghoffi* Hoffman, 1978	1	0	0
	*Aporodesmus* sp.	0	0	0
Cyrtodesmidae Cook, 1896	cyrtodesmid sp.	1	0	0
Fuhrmannodesmidae Brölemann, 1916	*Fuhrmannodesmus lividus* Carl, 1914	0	2	1
	fuhrmannodesmid sp.	0	2	1
	*Gyrophallus* sp.	0	2	1
Haplodesmidae Cook, 1895	*Prosopodesmus jacobsoni* Silvestri, 1910	1	0	0
Macrosternodesmidae Brölemann, 1916	*Ophiodesmus albonanus* (Latzel, 1895)	0	1	1
Nearctodesmidae Chamberlin and Hoffman, 1950	nearctodesmid sp.	0	2	1
Oniscodesmidae de Saussure, 1860	*Amphitomeus attemsi* (Schubart, 1934)	1	0	0
Opisotretidae Hoffman, 1980	*Corypholophus* sp.	0	2	?+
	*Solaenaulus butteli* (Carl, 1922)	0	2	+
Polydesmidae Leach, 1815	*Brachydesmus superus* (Latzel, 1884)	0	1	1
	*Propolydesmus laevidentatus* (Loksa, 1967)	0	1	1
Pyrgodesmidae Cook, 1895	*Rharodesmus tabarkensis* Akkari and Enghoff, 2011	1	0	0
	*Tonodesmus* sp.	1	0	0
	*Cynedesmus* sp.	1	0	0
	*Cryptocorypha ornata* (Attems, 1938)	1	0	0
Trichopolydesmidae Verhoeff, 1910	trichopolydesmid. sp.	0	2	1
	*Napocodesmus endogeus* Ceuca, 1974	0	2	1
Suborder Dalodesmidea Hoffman, 1980				
Dalodesmidae Cook, 1896	*Icosidesmus* sp.	0	1	1

**Table:** 

Char. no.→	1	2	3
Ammodesmidae	1	0	0
Cryptodesmidae	1	0	0
Cyrtodesmidae	1	0	0
Fuhrmannodesmidae	0	2	1
Haplodesmidae	1	0	0
Macrosternodesmidae	0	1	1
Nearctodesmidae	0	2	1
Oniscodesmidae	1	0	0
Opisotretidae	0	2	1
Polydesmidae	0	1	1
Pyrgodesmidae	1	0	0
Trichopolydesmidae	0	2	1
Dalodesmidae	0	1	1

## Appendix

List of characters and tentative character states

(1) Knobs on posterior surface of the prozonites: (0) absent; (1) present

(2) Shape of the limbus: (0) with one or two rows lobes additionally bearing simple spikes; (1) with ramified spikes and icicles; (2) reduced

(3) Rows of small intercalary scutes on metazonites: (0) absent; (1): present

## References

[B1] Adis J Golovatch SI Wilck L Hansen B (2000) On the identities of *Muyudesmus obliteratus* Kraus, 1960 versus *Poratia digitata* (Porat, 1899), with first biological observations on parthenogenetic and bisexual populations (Diplopoda: Polydesmida: Pyrgodesmidae). Fragmenta Faunistica 43: 149-170.

[B2] Akkari N Enghoff H (2011) *Rharodesmus* Schubart, 1960 – a tropical element in the North African fauna: a new species from Tunisia and notes on the family Pyrgodesmidae (Diplopoda: Polydesmida). Zootaxa 2985: 55-63.

[B3] Arndt E Enghoff H Spelda J (2008) Millipedes (Diplopoda) of the Canarian Islands: Checklist and key. Vieraea 36: 1-28.

[B4] Attems C (1940) Myriapoda 3. Polydesmoidea III. Fam. Polydesmidae, Vanhoeffeniidae, Cryptodesmidae, Oniscodesmidae, Sphaerotrichopidae, Periodontodesmidae, Rhachidesmidae, Macellolophidae, Pandirodesmidae. Das Tierreich 70: 1-577.

[B5] Bergholz NGR Adis J Golovatch SI (2004) New records of the millipede *Myrmecodesmus hastatus* (Schubart, 1945) in Amazonia of Brazil (Diplopoda: Polydesmida: Pyrgodesmidae). Amazonia 18(1/2): 157–161.

[B6] Golovatch SI (1996) Two new and one little-known species of the millipede family Pyrgodesmidae from near Manaus, Central Amazonia, Brazil (Diplopoda: Polydesmida). Amazonia 16: 325-336.

[B7] Golovatch SI (2003) A review of the volvatory Polydesmida, with special reference to the patterns of volvation (Diplopoda). African Invertebrates 44 (1): 39-60.

[B8] Golovatch SI Geoffroy J-J Mauriès J-P VandenSpiegel D (2009a) Review of the millipede family Haplodesmidae Cook, 1895, with descriptions of some new or poorly known species (Diplopoda, Polydesmida). ZooKeys 7: 1-53. 10.3897/zookeys.7.117

[B9] Golovatch SI Geoffroy J-J Mauriès J-P VandenSpiegel D (2009b) Review of the millipede genus *Eutrichodesmus* Silvestri, 1910 (Diplopoda, Polydesmida, Haplodesmidae), with descriptions of new species. ZooKeys 12: 1-46. 10.3897/zookeys.12.167 PMC4453233 26052236

[B10] Golovatch SI Geoffroy J-J Mauriès J-P VandenSpiegel D (2008) The first, new species of the millipede family Pyrgodesmidae to be recorded in Vanuatu, Melanesia, southwestern Pacific (Diplopoda: Polydesmida). Arthropoda Selecta 17(3–4): 145-151.

[B11] Golovatch SI Hoffman RL Knapinski S Adis J (2001) Review of the millipede genus *Cylindrodesmus* Pocock, 1889 (Diplopoda: Polydesmida: Haplodesmidae). Fragmenta Faunistica 44: 179-201.

[B12] Golovatch SI Semenyuk II VandenSpiegel D Ainchkin AE (2011) Three new species of the millipede family Pyrgodesmidae from Nam Cat Tien National Park, Southern Vietnam (Diplopoda: Polydesmida). Arthropoda Selecta 20 (1): 1-9.

[B13] Golovatch SI Sierwald P (2000) Review of the millipede genus *Poratia* Cook and Cook, 1894 (Diplopoda: Polydesmida: Pyrgodesmidae). Arthropoda Selecta 9 (3): 181-192.

[B14] Hoffman RL (1980) [for 1979] Classification of the Diplopoda. Muséum d’Histoire Naturelle Genève, Genève, 237 pp.

[B15] Mesibov R (2003) Two new and unusual genera of millipedes (Diplopoda: Polydesmida) from Tasmania, Australia. Zootaxa 368: 1-32.

[B16] Mesibov R (2005) A new genus of millipede (Diplopoda: Polydesmida: Dalodesmidae) from Tasmania with a pseudo-articulated gonopod telopodite. Zootaxa 1064: 39-49.

[B17] Mesibov R (2008) The millipede genera *Gephyrodesmus* Jeekel, 1983 and *Orthorhachis* Jeekel, 1985 in southeastern Australia, a new *Lissodesmus* Chamberlin, 1920 from Victoria, and observations on male leg setae, spinnerets and metatergite sculpture (Diplopoda: Polydesmida: Dalodesmidae). Zootaxa 1790: 1-52.

[B18] Mesibov R (2009a) New and little-used morphological characters in Polydesmida (Diplopoda). Soil Organisms 81 (3): 531-542.

[B19] Mesibov R (2009b) Revision of *Agathodesmus* Silvestri, 1910 (Diplopoda, Polydesmida, Haplodesmidae). ZooKeys 12: 87-110. 10.3897/zookeys.12.206 PMC3760138 24003320

[B20] Mesibov R (2009c) A new millipede genus and a new species of Asphalidesmus Silvestri, 1910 (Diplopoda, Polydesmida, Dalodesmidea) from southern Tasmania, Australia. ZooKeys 7: 55-74. 10.3897/zookeys.93.1255 PMC3095182 21594078

[B21] Mesibov R (2011) New species of *Asphalidesmus* Silvestri, 1910 from Australia (Diplopoda, Polydesmida, Dalodesmidea). ZooKeys 93: 43-65. 10.3897/zookeys.93.1255 PMC3095182 21594078

[B22] Rowe M Sierwald P (2006) Morphological and systematic study of the tribe Australiosomatini (Diplopoda: Polydesmida: Paradoxosomatidea: Paradoxosomatidae) and a revision of the genus *Australiosoma* Brölemann. Invertebrate Systematics 20: 527-556. 10.1071/IS05034

[B23] Schmidt D (1962) Über die taxonomische Wertigkeit von Structuren des Metazoni-Hinterrandes bei Diplopoden. Senckenbergiana biologica 43 (1): 65-80.

[B24] Shear WA (1973) Millipedes (Diplopoda) from Mexican and Guatemalan caves. Subterranean Fauna of Mexico, 2. Problemi attuali di scienza e di cultura, quaderno accademia nazionale dei Lincei 171: 239-305.

[B25] Shear WA (1977) Millipedes (Diplopoda) from Caves in Mexico, Belize and Guatemala III. Subterranean Fauna of Mexico, 3. Problemi Attuali di Scienza e di Cultura, Quaderno Accademia Nazionale dei Lincei 171 (3): 235-265.

[B26] Shear WA (2008) Spinnerets in the milliped order Polydesmida, and the phylogenetic significance of spinnerets in millipeds (Diplopoda). International Journal of Myriapodology (2): 123–146.

[B27] Shelley RM (2003) [for 2002] A revised, annotated, family-level classification of the Diplopoda. Arthropoda Selecta 11: 187-207.

[B28] Silvestri F (1925) Descripción de un nuevo género de Polydesmidae (Myriapoda, Diplopoda) de España meridional. Boletín de la Real Sociedad Española: 368–375.

[B29] Silvestri F (1947) Redescrizione del genere *Cynedesmus* O.F. Cook (Diplopoda, Polydesmoidea). Bollettino del Laboratorio di Entomologia Agraria: 93–96.

[B30] Simonsen A (1990) Phylogeny and biogeography of the millipede order Polydesmida, with special emphasis on the suborder Polydesmida. Thesis in Systematic Zoology 1990, University of Bergen Norway, 114 pp.

[B31] Vanden Spiegel D Golovatch SI (2003) Review of the East African millipede genus *Elassystremma* Hoffmann and Howell, 1081 (Diplopoda: Polydesmida: Ammodesmidae). Arthropoda Selecta 12(3–4): 183-191.

